# A Review of Therapeutic Strategies against Cardiac Fibrosis: From Classical Pharmacology to Novel Molecular, Epigenetic, and Biotechnological Approaches

**DOI:** 10.31083/j.rcm2408226

**Published:** 2023-08-08

**Authors:** Erica Floris, Claudia Cozzolino, Sangar Marconi, Fabiana Tonicello, Vittorio Picchio, Francesca Pagano, Isotta Chimenti

**Affiliations:** ^1^Department of Medical Surgical Sciences and Biotechnologies, Sapienza University, 04100 Latina, Italy; ^2^Institute of Biochemistry and Cell Biology, National Council of Research (IBBC-CNR), 00015 Monterotondo, Italy; ^3^Mediterranea Cardiocentro, 80122 Napoli, Italy

**Keywords:** cardiac fibrosis, cardiac remodeling, biological therapies, cardiac fibroblasts, cardiac stromal cells, non-coding RNAs, RNA-therapeutics, precision medicine

## Abstract

Cardiovascular diseases are the first cause of death worldwide, with a heavy social and economic impact. They include a wide range of pathological conditions, among which cardiac fibrosis represents a common pathogenetic hallmark. The fibrotic process is driven by cardiac mesenchymal stromal cells, namely fibroblasts, which become activated, proliferate, and differentiate into myofibroblasts in response to several stimuli, in the end secreting extracellular matrix proteins, and mediating cardiac tissue remodelling and stiffening. A specific therapy for the exclusive treatment of cardiac fibrosis is still lacking. Given the growing quest for reducing the burden of cardiovascular diseases, there is increasing interest in the search for new effective anti-fibrotic therapies. In this review, we will briefly summarize the limited pharmacological therapies known to act, at least in part, against cardiac fibrosis. Then we will present novel potential active molecules, molecular targets, and biotechnological approaches emerged in the last decade, as possible future therapeutic strategies for cardiac fibrosis, with a specific focus on targeting fibroblast activation and function.

## 1. Introduction

Heart disease is a leading cause of death worldwide and its prevalence is expected to increase in the next decades [1, 2, 3], representing a heavy social and economic burden globally. Cardiac fibrosis is one of the key underlying pathogenetic mechanisms of many chronic cardiac diseases, and its therapeutic management remains an unmet clinical need [4].

The fibrotic process develops either acutely as a circumscribed scar due for example to acute ischemia and cell death, or in the interstitium as a continuum positive feedback of multiple attempts for repair reactions. In this case, it evolves into a chronic degenerative/inflammatory response with an increasing number of mesenchymal stromal cells turning into activated cardiac fibroblasts (CFs). Under physiological conditions, most stromal cells (including CFs) are static, but when an injury occurs, CFs become activated, proliferate, and then secrete a large amount of collagen fibers, leading to extra-cellular matrix (ECM) resorption and deposition [5]. These phenomena will progressively increase myocardial stiffness, hamper myocardial conduction, reduce the efficiency of the cardiac muscle contraction, all of which will ultimately lead to heart failure [6, 7].

At present, there is no specific treatment targeting exclusively myocardial fibrosis. The increasing quest for novel effective drugs against fibrosis and remodelling respond to international research priorities for the development of precision and personalized medicine approaches to chronic non-communicable diseases in the aging population, which could reduce the cardiovascular disease burden effectively. Indeed, novel efficient anti-fibrotic drugs may reduce the cost of surgical-clinical interventions and of standard life-long medication, as well as the incidence and average time of hospitalization.

In this review, we will provide a brief overview of the current medications known to interfere, at least in part, with the mechanisms of cardiac mesenchymal and fibroblast activation and function. Then we will present a selection of novel potentially active molecules, whose mechanism of action is not necessarily fully elucidated yet, together with a review of novel possible molecular targets in CF activation. We will also present a selection of epigenetic pathways and non-coding RNAs known to interfere directly with fibroblast differentiation. Finally, we will present some examples of biological and biotechnological approaches (e.g., direct reprogramming, T-cell immunotherapy) that have been recently described against cardiac fibrosis.

## 2. Consolidated Pharmacological Targets

### 2.1 The Renin-Angiotensin-Aldosterone System 

Cardiac fibrosis development is directly associated with the activation of the renin-angiotensin-aldosterone system (RAAS) [8]. The progression of cardiac dysfunction reduces renal perfusion, so the juxtaglomerular apparatus starts to release renin [9]. The consequence of this release is an increment of the angiotensin II level produced by the angiotensin-converting enzyme (ACE). For a long time, angiotensin II has been considered the principal promoter of cardiac fibrosis [10]. In fact, angiotensin II directly drives numerous myofibroblast functions such as, cell proliferation and migration, transforming growth factor beta (TGF-β) release, and ECM synthesis through stimulation of the angiotensin II type 1 receptor (AT1R) [11, 12, 13, 14]. The anti-fibrotic mechanisms triggered by RAAS inhibition have been widely described in the literature through animal studies, together with the direct role of angiotensin II infusion in enhancing cardiac fibrosis in rodents [15]. To date, ACE inhibitors and AT1R antagonists represent the mainstay therapy in heart failure patients. They act in multiple ways by reducing ECM production and cardiac hypertrophy, as demonstrated both in cell culture and animal models [16, 17, 18]. Nonetheless, it was demonstrated that the specific activation of AT1R in cardiomyocytes has only a minimal impact on cardiac hypertrophy, suggesting a main role of this receptor in cardiac non-myocytic cells [19].

Aldosterone is a steroid hormone produced by adrenal cortex under stimulation of angiotensin II [20]. It is a transcriptional regulator and is responsible for upregulating pro-fibrotic genes. Aldosterone and angiotensin II work together by activating the mitogen-activated protein kinase (MAPK) pathway in cardiomyocytes [21, 22], as well as in CFs [23, 24]. Interestingly, aldosterone can exert its profibrotic effect also when angiotensin II is inactivated, suggesting its independent role in promoting fibrosis [25]. There are two drugs—spironolactone and eplerenone—which have revealed potential antifibrotic effects clinically. They are receptor antagonists of the mineralocorticoid family, blocking aldosterone signaling. Zannad *et al*. [26] verified the serum level reduction of fibrosis markers and collagen synthesis after spironolactone treatment. More data on their specific effect on CFs is needed, though.

### 2.2 Beta-Adrenergic Signaling

Pharmacological approaches for the treatment of cardiac fibrosis include targeting the adrenergic receptor system, as well. This neurohormonal pathway contributes to the maintenance of cardiovascular homeostasis through the release of norepinephrine, a neurotransmitter that binds to both type α adrenergic receptors (α-ARs) on the peripheral vasculature, or type β1 adrenergic receptors (β1-ARs) mostly on cardiomyocytes, with positive inotropic and chronotropic effects [20]. However, under chronic pathological conditions, continuous activation of ARs in the heart leads to maladaptive effects, such as adverse remodelling of cardiac tissue. In these cases, one of the most effective therapies to prevent heart failure is the blockade of β1-ARs on cardiomyocytes through β-blockers drugs [27, 28, 29], which have a well-established and consolidated efficacy. Beta-1 adrenergic signaling, though, has been shown to exert a direct effect also on human cardiac mesenchymal stromal cells, increasing gene expression of fibrotic markers such as collagen type I alpha 1 (*COL1A1*) and thymus cell antigen 1 (*THY1*). Indeed, the cells isolated from patients undergoing treatment with β-blockers displayed reduced expression of markers of fibrotic activation, and a less fibrotic epigenetic profile, compared to cells derived from patients not following a β-blockers therapy regimen [30].

Other types of β-ARs are expressed in cardiac non-myocytes, which play different roles. In detail, CFs mainly express β2-ARs, but their role in cardiac fibrosis pathways is still debated. Studies performed over the last decade have reported conflicting results, showing both pro- and anti-fibrotic responses [31]. Several studies have demonstrated that the activation of the β2-AR signalling in CFs inhibits the production of collagens and the transition to myofibroblasts, even under pro-fibrotic stimuli [32, 33, 34]. Other anti-fibrotic interventions have focused on the enhancement of the β2-AR signalling pathway via inhibiting the G protein-coupled receptor kinase 2 (GRK2), which phosphorylates the receptor leading to its desensitization. Different works [35, 36, 37] have also described that the fibroblast-specific inhibition of GRK2 could prevent cardiac fibrosis and dysfunction in animal models of heart failure. Nonetheless, β2-ARs have been shown to increase DNA synthesis, proliferation, and production of pro-fibrotic interleukin 6 (IL-6) in isolated rat and human CFs [38, 39, 40]. Conversely, specific β2-blockade in human mesenchymal stromal cells could reduce *THY1* and *COL1A1* expression, and the release of several pro-inflammatory cytokines [41]. Further studies are required on this pathway to understand the reasons for these conflicting results and hopefully reach a consensus.

Besides β1- and β2-ARs, a third type of adrenergic receptor is expressed in cardiac tissue, namely the β3-AR, whose levels are low in healthy hearts, but become up-regulated under pathological conditions with anti-fibrotic effects [42]. The potential advantage of fibrosis targeting through β3-AR-based approaches is that these receptors cannot be phosphorylated by GRKs, thus being resistant to desensitization [43]. Several studies have demonstrated the beneficial effects of β3-AR agonists on preventing myocardial fibrosis in animal models of myocardial infarction (MI) and pressure overload [44, 45]. Interestingly, a β3-AR-selective agonist, Mirabegron, has already obtained food and drug administration (FDA) approval for the treatment of overactive bladder, and it is now under assessment for the treatment of hypertensive structural heart disease [46].

## 3. Novel Potential Active Molecules

Many molecules have been described in the literature as able to directly act on cardiac stromal cells and fibroblasts, reducing fibrotic activation and features. In some cases, the signalling pathways involved have been defined, although for many of them detailed insights on signal transduction and molecular mechanisms are still lacking or incomplete (Table 1, Ref. [47, 48, 49, 50, 51, 52, 53, 54, 55, 56, 57, 58, 59, 60, 61, 62]). Nonetheless, they represent an important starting point for further validation and translational studies.

**Table 1.** **Novel potential active molecules against cardiac fibroblast activation or function for the treatment of cardiac fibrosis**.

Molecule	Target	Mechanism of action	Model system	References
Vinpocetine	PDE1	Activation of NF-κB (also through IKK) with anti-inflammatory effects.	*In vitro*	[47]
			*In vivo*	
IC86340	PDE1A	Reduced fibroblast activation, ECM deposition, and expression of pro-fibrotic genes.	*In vitro*	[48]
			*In vivo*	
TP-10	PDE10A	Reduced cardiac remodeling and fibrosis.	*In vivo*	[49]
Dapagliflozin	SGLT2	AMPKα-mediated inhibition the TGF‐β/SMAD pathway, reducing proliferation, activation, and collagen production of CFs.	*In vitro*	[59]
			*In vivo*	
Berberine	IGF-1R	Downregulation of IGF-1R activation, reducing MMP2, MMP9, α-SMA, and collagen I.	*In vitro*	[50]
Naringenin	ERK, JNK, PI3K/Akt, SMAD3	Inhibition of TGF-β-induced proliferation and collagen production in fibroblasts, suppression of *COL1A1*, *COL3A1*, *ACTA2*.	*In vitro*	[51, 52]
Bufalin and lycorine	Unclear/multiple	Suppression of collagen I and matrix components in activated fibroblasts.	*In vitro*	[58]
Piperlongumine	Unclear/multiple	Reduced expression and transcriptional activity of KLF4 in fibroblasts.	*In vivo*	[53]
		Reduced phosphorylation of Akt and preserved levels of FoxO1 in cardiomyocytes.		
Curcumin	Unclear/multiple	Activation of SIRT1 with reduced MMP2 and 9, collagen I and III levels in fibroblasts. Reduced IL-18 secretion and TGF-β-p-SMAD2/3 activation in fibroblasts. Increased SMAD7, leading to TGF-β signaling attenuation. Inhibition of mTOR signaling thus enhancing autophagy.	*In vivo*	[54, 55, 56]
Trehalose	Autophagy	Reduced expression of markers of activated fibroblasts, and reduced fibrotic and inflammatory paracrine signaling. Increased pro-angiogenic paracrine support.	*In vitro*	[57]
MCC950	NLRP3 complex	Blocking of TGF-β/SMAD4 pathway, with reduced α-SMA levels, cardiac inflammation and macrophage infiltration. Reduced calcineurin expression and MAPK activation, attenuating cardiac hypertrophy.	*In vivo*	[60]
pUR4	Fibronectin polymerization	Reduced cell proliferation and migration, and ECM deposition. Reduced remodeling and fibrosis.	*In vitro*	[61]
			*In vivo*	
Verteporfin	YAP/TAZ-TEADs complex	Downregulation of the genes induced by TGF-β. Reduced fibrosis and remodeling.	*In vitro*	[62]
			*In vivo*	

PDE, phosphodiesterase; SGLT2, sodium-glucose cotransporter-2; IGF-1R, insulin-like growth factor 1 receptor; ERK, extracellular signal-regulated kinase; JNK, c-Jun N-terminal kinase; PI3K/Akt, phosphatidylinositol3-kinase/protein kinase B; SMAD, small mothers against decapentaplegic; NLRP3, NOD-like receptor family pyrin domain containing 3; YAP/TAZ, Yes-associated protein/transcriptional coactivator with PDZ-binding motif; TEADs, TEA domain transcription factors; ECM. extra-cellular matrix; TGF-β, transforming growth factor beta; *COL1A1*, collagen type I alpha 1; *COL3A1*, collagen type III alpha 1; *ACTA2*, actin alpha 2; α-SMA, alpha smooth muscle actin; MMP, matrix metalloproteinase; KLF4, Krüppel-like factor 4; SIRT1, sirtuin1; NF-κB, nuclear factor kappa B; IKK, IkappaB kinase; CFs, cardiac fibroblasts.

### 3.1 Natural Compounds and Nutraceuticals

Some active molecules occur naturally in food and could be used in clinics due to the wide range of pharmacological effects.

Vinpocetine is derived from a natural plant alkaloid and is used as a dietary supplement. It appears to act through an IkappaB kinase (IKK)-dependent pathway, attenuating CF activation and ECM synthesis, independently from small mothers against decapentaplegic (SMAD) phosphorylation or activation [47, 63]. Moreover, both *in vitro* and *in vivo*, it targets cyclic nucleotide phosphodiesterase 1 (PDE1), whose expression is up-regulated in the heart under RAAS and beta-adrenergic stimulation [48, 64]. Vinpocetine could also act through IKK with anti-inflammatory effects because of its role in nuclear factor kappa B (NF-κB) activation [47], suggesting the potential to contribute to anti-remodelling effects [65].

Concerning cyclic nucleotide PDEs as promising targets for the modulation of the fibrotic process, the PDE1A form has been identified as a key regulator of fibroblast activation and ECM remodelling in the heart [48]. This isoform was found to be specifically enhanced in activated myofibroblasts both *in vitro* upon angiotensin II and TGF-β stimulation, and *in vivo* in mouse, rat, and human fibrotic hearts. Miller *et al*. [48] demonstrated that the PDE1-selective inhibitor IC86340 reduces activation, ECM deposition, and the expression of pro-fibrotic genes in rat CFs, as well as the levels of alpha smooth muscle actin (α-SMA), collagen I, and plasminogen activator inhibitor I (PAI-I) levels, all markers of cardiac fibrosis. *In vivo*, isoproterenol-induced interstitial fibrosis was attenuated upon PDE1A inhibition in mouse hearts [48]. Another PDE family member involved in pathological cardiac remodelling is PDE10A, identified by Chen and colleagues [49] as abnormally expressed in human and mouse failing hearts, as well as in murine CFs stimulated with TGF-β. Cardiac remodelling and fibrosis in angiotensin-infused mice were attenuated by specific inhibition of PDE10A through the administration of the selective inhibitor TP-10 [49]. Interestingly, PDE10A is an already known safe druggable target, since several PDE10A inhibitors have already passed phase I clinical trials in humans for the treatment of schizophrenia [66, 67] and Huntington’s disease [68, 69].

Berberine is derived from a natural plant alkaloid able of targeting the insulin-like growth factor I receptor (IGF-1R). IGF-1 is an important mediator of cardiac hypertrophy, but its continuous expression in the myocardium contributes to pathological remodelling and interstitial fibrosis [70]. The IGF-1R is upregulated in CFs isolated from diabetic hearts, characterized by overexpression of matrix metalloproteinase matrix metallopeptidase-2 (MMP-2) and matrix metallopeptidase-9 (MMP-9), α-SMA, and collagen I. The long-term treatment with berberine has demonstrated its capacity to downregulate IGF-1R and all its downstream effects in a diabetic rat model [50].

Naringenin, a flavonoid present in citrus fruits, exerts a cardioprotective effect by suppressing extracellular signal-regulated kinase (ERK), c-Jun N-terminal kinase (JNK), and phosphatidylinositol3-kinase/protein kinase B (PI3K/Akt) signalling pathways. *In vitro*, naringenin caused the inhibition of TGF-β-induced proliferation and collagen production in CFs, partly mediated by arresting DNA synthesis at G0/G1 [51]. A recent study proposed a further mechanism targeting cardiac fibrosis through naringenin. In this work, it suppresses the expression of profibrotic proteins such as collagen I, collagen III, and α-SMA by inactivating the SMAD3 signalling pathway in angiotensin-stimulated mouse CFs. In addition, naringenin acts as a SMAD3-inhibitor, and was shown to reduce the proliferation and ECM production by CFs in rats with hypertension-induced atrial fibrosis [52].

Piperlongumine is another alkaloid that is naturally isolated from the long pepper piper, and it has been proposed as a potential natural compound for the clinical treatment of cardiac hypertrophy and fibrosis. Angiotensin-induced expression of profibrotic proteins in neonatal CFs was significantly decreased by piperlongumine treatment, reducing the expression of Krüppel-like factor 4 (KLF4), and its recruitment to the promoter regions of the pro-fibrotic cytokines TGF-β and connective tissue growth factor (CTGF). Like many other therapeutic strategies, the effects of piperlongumine are not restricted to non-myocytes. In fact, treatment with piperlongumine exerted also an anti-hypertrophic effect in neonatal rat cardiomyocytes by reducing the phosphorylation of Akt, thereby preserving the level of Forkhead box transcription factor O1 (FoxO1) [53]. The FoxO protein family, in fact, plays an important role in various biological processes such as metabolism, apoptosis and oxidative stress [71], blunting pathological hypertrophy through the inhibition of the calcineurin/nuclear factor of activated T-cells (NFAT) pathway [72]. Thus, piperlongumine could be considered as a preventive strategy, as well.

Curcumin is a spice-derived pigment, widely studied as an anti-cancer, anti-oxidant, and anti-inflammatory agent [73, 74]. Recently, it was investigated also as a modulator of cardiac fibrosis. Hsu and colleagues [75] reported reduction of TGF-β in mice with MI treated with curcumin, describing modulation of sirtuin1 (SIRT1) activity. SIRT1 is a conserved histone deacetylase involved in various biological processes [75]. Recently, several pieces of evidence support the role of SIRT1 in cardiac fibrosis, as well [76]. Treatment with curcumin led to the activation of SIRT1 and to the decrement of metalloproteases 2 and 9, and collagen I and III levels in angiotensin-treated CFs [54]. Moreover, curcumin affected macrophage-fibroblast crosstalk in a mouse model of MI, reducing pro-inflammatory signalling by macrophages. In turn, IL-18 expression and secretion by CFs were reduced, as well as the activation of the TGF-β-p-SMAD2/3 network [77]. Other studies have highlighted how curcumin also increases SMAD7, which is a direct inhibitor of SMAD2/3 phosphorylation and a disruptor of the SMAD-complex formation [78], further leading to the attenuation of pro-fibrotic TGF-β signalling [55].

A recent study reported the ability of curcumin to also modulate autophagy. In this case, it inhibited mammalian target of rapamycin (mTOR) signalling thus enhancing the autophagic process. Consequently, all pro-fibrotic markers such as procollagen I and III in rat hearts treated with isoprenaline were reversed by curcumin administration [56]. However, whether this *in vivo* effect was due to direct or indirect mechanisms has not been defined yet.

The disaccharide trehalose—derived from myocytes—is known to be a natural activator of autophagy. It has shown protective effects on the heart [79], and can specifically enhance autophagy also in resident cardiac mesenchymal stromal cells [80] promoting anti-fibrotic responses. Autophagy boosting in these cells can reduce the expression of markers of activated fibroblasts, and preserve a less pro-fibrotic and pro-inflammatory paracrine function. When cells are exposed to metabolic stress, which is a main mediator of myocardial fibrosis, trehalose treatment also increased pro-angiogenic effects of cardiac mesenchymal stromal cells [57]. This effect has been also confirmed in human cells derived from diabetic patients, where the enhancement of autophagy by trehalose, combined with oxidative stress reduction by nitrogen oxides (NOX)-inhibitors, may contribute to reducing fibrotic activation and specification of primitive stromal cells [81].

A recent study by Schimmel *et al*. [58] has identified fifteen substances with antiproliferative effects in human CFs by high-throughput natural compound library screening. The validation by multiple *in vitro* fibrosis assays and selection by stringent algorithms have identified the steroid bufalin and the alkaloid lycorine as potentially effective antifibrotic molecules. Both were assayed for suppression of collagen I and matrix components production in activated fibroblasts, and the most significant effects were mediated by bufalin [58].

### 3.2 Synthetic Drugs and Peptides

Dapagliflozin is a sodium-glucose Cotransporter-2 (SGLT2) inhibitor. SGLT2 is involved in the pathogenetic mechanisms of diabetes by increasing the risk of cardiovascular disorders, such as MI, stroke, and heart failure [82]. The development of diabetic cardiomyopathy is caused by functional and metabolic changes that occur in the myocardium at multiple levels [57]. In the last years, studies have shown how SGLT2 inhibitors exhibit cardiovascular safety and benefits [83]. Dapagliflozin blocks glucose resorption in the proximal tubule of the kidney and promotes glucosuria [84, 85, 86], but it also mediates antifibrotic effects in an AMPKα-dependent manner. The AMPKα activity reduction has been observed in failing human and animal hearts, and it correlates to cardiac fibrosis [87, 88]. At the molecular level, it was shown that AMPKα activation mediated by dapagliflozin inhibits the up-regulation of the TGF-β/SMAD pathway detected in diabetic hearts, reducing proliferation, activation, and collagen production of CFs when stimulated with high glucose [59].

MCC950 is a selective inhibitor of the inflammasome complex NOD-like receptor family pyrin domain containing 3 (NLRP3), that has been proposed to reverse transverse aortic constriction (TAC)-induced cardiac remodelling. It is known that the prevention of myocardial remodelling is possible by suppressing the expression or activation of NLRP3 before TAC surgery [89]. Zhao *et al*. [60] have reported that NLRP3 activation promotes pathological cardiac remodelling due to pressure overload in a mouse model, whereas MCC950 treatment significantly attenuated fibrosis. Specifically, it reduced α-SMA levels, blocked the TGF-β/SMAD4 pathway, and reduced cardiac inflammation, and macrophage infiltration. At the same time, MCC950 reduced calcineurin expression and MAPK activation, affecting and attenuating cardiac hypertrophy both around peripheral blood vessels and in the myocardial interstitium [60]. The action of MCC950 is exerted also on the microenvironment by inhibiting the release of inflammatory factors IL-18 and IL-1β. This evidence was studied *in vivo* on MCC950-treated post-MI mice, and the marked reduction of collagen I, collagen III, and α-SMA levels was clear. The reduction of inflammatory cell infiltration was shown by the expression of cleaved IL-1β and IL-18 decrement. These findings confirm the multi-pathway action of this drug in opposing cardiac fibrosis, partly mediated by suppressing the microenvironment inflammation [90].

A new strategy proposed to prevent the development of cardiac fibrosis is the intervention on ECM protein polymerization. Specifically, the small peptide pUR4, a fibronectin polymerization inhibitor, has been tested in mouse models of ischemia/reperfusion (I/R) injury to attenuate pathological remodelling, tissue fibrosis, and cardiac dysfunction. *In vitro*, pUR4 administration to murine and human myofibroblasts led to a reduction of cell proliferation and migration, as well as ECM protein deposition, thus ameliorating pathological features [61].

A recent study aimed to demonstrate how pharmacological interference with mechano-sensing cues could oppose stromal cell activation and myofibroblast differentiation. Indeed, mechano-sensing signalling due to myocardial stiffening plays a key role in remodelling progression. In particular, the shuttling of the main Hippo transcriptional component, the YAP (Yes-associated protein)/TAZ (transcriptional coactivator with PDZ-binding motif) complex, exerts homeostatic control of cardiac matrix, and a specific function in myocardial remodelling after injury [91, 92]. Garoffolo *et al*. [62] tested treatment with Verteporfin, a drug known to prevent the association of the YAP/TAZ complex with their cognate transcription factors TEA domain transcription factors (TEADs), on human fibroblasts activation and differentiation. The results revealed prevention of the fibrotic process by significant downregulation of more than half of the genes induced by TGF-β exposure. Verteporfin was also tested *in vivo* in mice with permanent cardiac ischemia, significantly reducing indexes of fibrosis and morphometric remodelling [62]. In addition, Francisco and colleagues [63] have demonstrated that YAP activation in CFs triggers cell proliferation and expression of pro-fibrotic genes through the myocardin related transcription factor A (MRTF-A) transcriptional coactivator. Inducing YAP deletion in fibroblasts also prevented myofibroblast transition, and the development of cardiac fibrosis and dysfunction in mouse models. These findings further emphasize the potential of targeting YAP as a strategy for anti-fibrotic therapies [63].

It is worth mentioning that the expanding knowledge on the mechanism of action of commonly prescribed drugs may represent a starting point as repurposing strategy towards cardiac fibrosis. Statins are the most common drugs used to inhibit cholesterol biosynthesis. Recently, several studies have demonstrated their capability of exerting other beneficial effects on cardiovascular diseases [93, 94]. Specifically, atorvastatin was studied as a potential drug to ameliorate cardiac fibrosis. It was used to treat CFs *in vitro* and achieved reduction of Heat Shock Protein 47 (HSP47) levels. Indeed, HSP47 is a collagen specific molecular chaperon, normally induced by TGF-β. *In vivo*, treated Dahl salt-sensitive rats showed indexes of cardiac fibrosis reduction associated with improvement of diastolic function after atorvastatin administration [95].

## 4. New Possible Molecular Targets

**Fig. 1.**
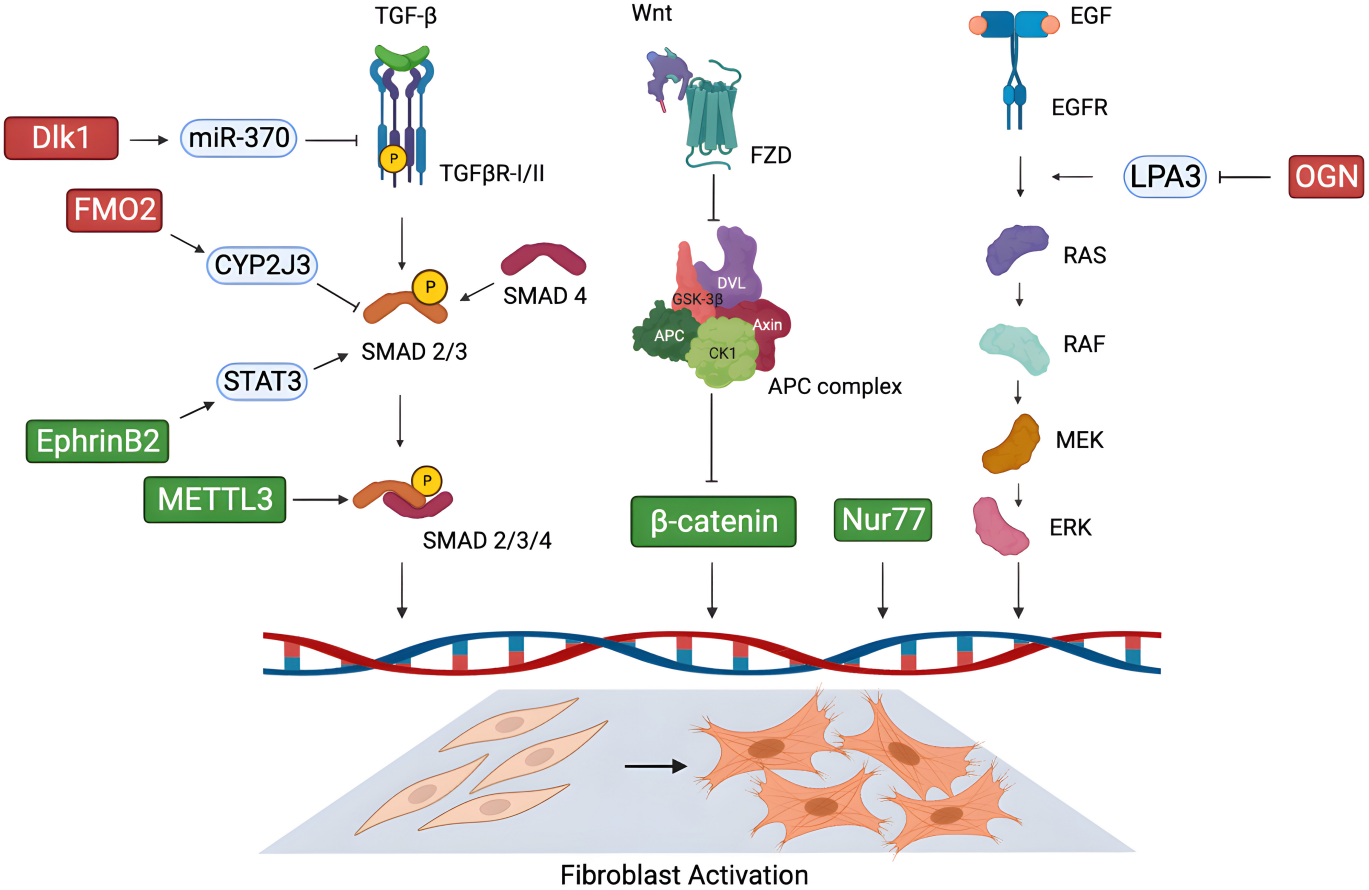
**Novel possible molecular targets for cardiac fibrosis**. New potential targets proposed in the literature in recent years for the treatment of cardiac fibrosis, and their mechanism of action in modulating cardiac fibroblast activation through key signalling pathways. (Green: pro-fibrotic factors; red: anti-fibrotic factors). Figure was created with the Biorender Software (https://www.biorender.com/). Dlk1, delta-like homolog 1; FMO2, flavin-containing monooxygenase 2; CYP2J3, cytochrome P450 superfamily 2J3; STAT3, signal transducer and activator of transcription 3; EphrinB2, erythropoietin-producing hepatoma interactor B2; METTL3, methyltransferase-like 3; TGF-β, transforming growth factor-beta; TGFβR, transforming growth factor-beta receptor; SMAD, small mothers against decapentaplegic; Wnt, wingless-INT; FZD, Freezled; APC, anaphase-promoting complex; Nur77, nuclear receptor 77; EGF, epidermal growth factor; EGFR, epidermal growth factor receptor; LPA3, lysophosphatidic acid 3; OGN, osteoglycin; RAS, rat sarcoma; RAF, rapidly accelerated fibrosarcoma; MEK, mitogen-activated protein kinase kinase; ERK, extracellular signal-regulated kinase; DVL, dishevelled; CK1, casein kinase; GSK-3β, glycogen synthase kinase-3 beta.

Besides the discovery and testing of novel molecules with one or more known targets, in the last decades many studies have focused on the identification of new potential molecular targets for cardiac fibrosis (Fig. 1), yet in the absence of known modulators.

Osteoglycin (OGN) is a small leucine-rich proteoglycan secreted in the ECM, whose role in the fibrotic process is still debated. OGN was found to be overexpressed in the heart of patients with hypertensive [96] and ischemic disease [97]. In 2018, Zuo *et al*. [98] identified OGN as a negative regulator of cardiac fibrosis in response to chronic hypertension-induced injury. Angiotensin II infusion in OGN deficient (OGN-/-) mice revealed increased interstitial fibrosis and diastolic dysfunction. Accordingly, *in vitro* angiotensin II treatment on OGN-/- CFs led to enhanced cell proliferation and migration via activation of the epidermal growth factor receptor (EGFR) signalling, while OGN overexpression had the opposite effect. Specifically, the authors demonstrated that in physiological conditions OGN attenuates cardiac fibrosis binding to lysophosphatidic acid 3 (LPA3), thus preventing the activation of the Rho-dependent EGFR/ERK signalling, which induces fibroblast proliferation and migration [98].

Another interesting modulator of cardiac fibrosis is the nuclear receptor Nur77. It is involved in stress responses in different cell types [99, 100, 101], and it seems to play a dual role in the fibrotic process in heart tissue. A study by Medzikovic *et al*. [102] highlighted how Nur77 promoted CF differentiation into myofibroblasts. Conversely, its knockdown led to a reduction of myofibroblast marker expression and ECM proteins production, as well as decreased proliferation and wound healing capacity in rat cells stimulated with isoproterenol or TGF-β. On the other hand, authors showed that Nur77 in cardiomyocytes prevented these cells from releasing paracrine factors involved in the fibroblast-to-myofibroblast transition, thus counteracting also indirectly the fibrotic response in the myocardium through intercellular communication [102].

Among the factors that take part in the fibrotic process, it is worth mentioning fine regulation of fibrosis-related gene expression carried out by the N6-methyladenosine transferase methyltransferase-like 3 (METTL3) at the post-transcriptional level. METTL3 is the catalytic subunit of the methyltransferase complex which mediates mRNA methylation [103, 104, 105]. Recently, Li *et al*. [106] discovered the role of METTL3 as a promoter of CF activation and ECM remodelling in the process of MI-induced fibrosis. Specifically, METTL3 exerted its pro-fibrotic properties via tuning the m6A modification of mRNAs encoding collagens and factors of the TGFβ/SMAD pathway [106]. METTL3 silencing significantly reduced the levels of collagens in a mouse model of coronary artery ligation, as well as proliferation and myofibroblast transition of TGFβ-stimulated CFs *in vitro*.

In 2019, delta-like homolog 1 (Dlk1), a member of the epidermal growth factor (EGF)-like family involved in the control of cell differentiation [107], has been identified as a key anti-fibrotic factor in the heart. In detail, authors found that Dlk1 negatively regulated fibroblast-to-myofibroblast differentiation through the activation of miR-370, which in turn inhibited the TGF-β receptor 2 signalling pathway in these cells. Indeed, Dlk1-null mice exhibited alteration of ECM composition, with increased collagen deposition, as well as cardiac dysfunction. In addition, Dlk1-null CFs showed hyperactivation of TGFβ/SMAD3 signalling and myofibroblast differentiation. Since Dlk1 expression was significantly reduced in fibrotic tissues from both human ischemic patients and infarcted pigs, Rodriguez and colleagues [108] hypothesized the potential positive targeting of this molecule for the treatment of cardiac fibrosis characterized by aberrant TGFβ-signalling activation.

Another factor whose pro-fibrotic activity has been recently uncovered is erythropoietin-producing hepatoma interactor B2 (EphrinB2), a cell surface transmembrane ligand ubiquitously expressed in mammals, mediating organ-specific regulation of the fibrotic process [109, 110]. Increased expression of EphrinB2 has been detected in human and mouse hearts with severe cardiac fibrosis. *In vivo* silencing of EphrinB2 led to the attenuation of cardiac fibrosis and improvement of cardiac function, while its overexpression in CFs mediated myofibroblast differentiation and activation *in vitro*. In detail, EphrinB2 exerted its pro-fibrotic effects mainly via activating the signal transducer and activator of transcription 3 (STAT3) signalling in CFs, and promoting the interaction between STAT3 and SMAD3, thus inducing the fibrotic response [111]. Given this evidence, EphrinB2 could be considered as another promising candidate target against cardiac fibrosis.

Recently, a previously unknown anti-fibrotic activity of flavin-containing monooxygenase 2 (FMO2) has been characterized by Ni *et al*. [112]. FMO2 is an enzyme with nicotinamide adenine dinucleotide hydrogen/flavin adenine dinucleotide (NADH/FAD)-dependent activity, whose expression was found specifically enriched in CFs, but not in cardiomyocytes, and significantly down-regulated upon cardiac injury in rats, mice, and non-human primates. FMO2 silencing in rat hearts induced spontaneous fibrosis and altered cardiac function; in contrast, induction of FMO2 expression in infarcted rat and monkey hearts attenuated pathological remodelling and cardiac dysfunction. Authors demonstrated that FMO2 inhibited SMAD2/3 activation through an enzymatic activity-independent way: mechanistically, FMO2 was found to bind to cytochrome P450 superfamily 2J3 (CYP2J3), thus preventing the interaction of the latter with SMURF2, an E3 ubiquitin ligase, which selectively mediates the proteasome-dependent degradation of phosphorylated SMAD2/3 in the nucleus [113]. Thus, FMO2 inhibits TGFβ-signalling in CFs through a conserved mechanism in human cells, paving the way for new therapeutic opportunities for cardiac fibrosis [112].

The Wnt/β-catenin signalling pathway plays a consolidated role in the fibrotic process by promoting the activation of CFs [114, 115, 116]. Xiang and colleagues [117] have investigated the effect of inducible β-catenin loss in CFs in mice undergoing TAC. Fibroblast-specific β-catenin depletion in this system revealed reduced interstitial fibrosis, improved cardiac function, as well as lower levels of cardiomyocyte hypertrophy. Although β-catenin loss did not affect the number of activated and differentiated CFs, the expression of many pro-fibrotic genes, such as *COL1A1*, collagen type III alpha 1 (*COL3A1*), periostin (*POSTN*), was significantly reduced [117]. Given its many roles in physiology and pathology, though, also related to epithelial-to-mesenchymal transition, targeting of this pathway for cardiac fibrosis will have to be highly organ-specific to avoid potential side effects.

Vainio *et al*. [118] has explored the effects of monoclonal antibody (mAb) therapy against connective tissue growth factor (CTGF), whose increased expression has been correlated with fibrotic diseases of virtually every human organ [119], including the heart [120]. CTGF-mAb administration in a mouse model of MI resulted in reduced cardiac fibrosis and hypertrophy, as well as increased survival and left ventricular function. Heart tissue also showed increased expression of genes associated with developmental and repair processes, and reduction of inflammatory and fibrotic genes. Mechanistically, CTGF-mAb treatment on human CFs *in vitro* attenuated the expression and production of α-SMA and collagen I following TGF-β stimulation, through the activation of the JNK2 pathway [118].

A new interesting potential target for the treatment of cardiac fibrosis has been identified by Schafer and colleagues [121]. In this study, RNA-sequencing analysis of human CFs stimulated *in vitro* with TGF-β identified interleukin 11 (IL-11) expression as the most positively correlated response during myofibroblast differentiation, while IL-11 levels in healthy tissues and cells were found to be unaffected [122, 123]. Authors found that IL-11 and its receptor IL11RA were expressed specifically in CFs in a mouse model of fibrotic heart. They demonstrated that IL-11 binds to soluble receptor IL11RA and mediates trans-signalling on other CFs. This promotes an autocrine effect which induces the production of fibrogenic proteins in an ERK-dependent way. *In vivo* overexpression of IL-11 was shown to induce cardiac fibrosis, while its depletion had a protective effect. Given this evidence, IL-11 could represent a promising target for new anti-fibrotic therapies [121].

## 5. Epigenetic Approaches

In the search for new potential therapeutic targets against cardiac fibrosis, the field of epigenetic approaches opens the possibility of fine tuning the activation and the progression of the fibrotic process. In the wide scenario of epigenetic regulators and non-coding RNAs, we briefly present examples of potential targets recently identified for therapeutic intervention in this pathological condition, focusing on those with proven specific activity in CFs.

### 5.1 Histone Modifications

In recent years, numerous studies have suggested that epigenetic regulation, including histone methylation, has extensive roles in fibrosis [124]. Wang *et al*. [125] studied how the lysine demethylase 5B (KDM5B), a histone H3K4me2/me3 demethylase, can affect the pathophysiological events in cardiac fibrosis. Initially, they demonstrated KDM5B upregulation in CFs in response to pathological stress. Secondly, it was proposed the molecular mechanism through which KDM5B exerts its action: it demethylates the activated H3K4me2/3 modification bounding the activating transcription factor 3 (Atf3) promoter, inhibiting its expression. In the literature [126] Atf3 is considered an antifibrotic regulator of cardiac fibrosis, and its suppression is correlated with the activation of TGF-β signalling. Finally, the authors prevented the fibroblast transition into myofibroblast by inhibiting KDM5B *in vitro* and *in vivo*, thus proposing KDM5B as possible candidate target to ameliorate cardiac fibrosis and remodelling [125].

Another fundamental epigenetic modulation is histone acetylation/deacetylation, that enhances or opposes the accessibility of transcriptional factors, respectively. It is known that small molecules acting as histone deacetylase (HDAC) inhibitors play a role in the attenuation of cardiac remodelling and fibrosis [127]. Nural-Guvener *et al*. [128] proposed a strategy through selective class I-HDAC inhibition against CFs activation. In particular, they treated cluster of differentiation 90-positive (CD90+) CFs *in vitro* with mocetinostat (an HDAC inhibitor), showing reduction of collagen III and α-SMA. Moreover, they demonstrated the efficacy of selective class I-HDACs inhibition in reducing fibrosis in a congestive heart failure animal model, as well as its effect in reversing interstitial fibrosis and improving cardiac function after MI [128].

In a previous study, it was demonstrated that a group of epigenetic reader molecules called bromodomain and extra-terminal (BET) acetyl-lysine binding proteins, play a crucial role in regulating pathological fibrosis [129]. In particular, Stratton and Bagchi *et al*. [130] identified by RNA-sequencing the bromodomain-containing protein 4 (BRD4) involvement in cardiac fibrosis promoting the activation of quiescent CFs into Periostin (Postn)-positive cells. The authors validated the pro-fibrotic role of BRD4 using the BET bromodomains inhibitor JQ1. Specifically, cultured primary adult rat ventricular fibroblasts stimulated with TGF-β in the presence of JQ1 showed reduced expression of fibrotic markers, such as α-SMA [130]. Moreover, mice were treated with the JQ1 inhibitor after transverse aortic constriction, and the transcriptomic profiling of the CFs isolated from the hearts suggested a significant downregulation of Postn mRNA expression.

### 5.2 Long Non-Coding RNAs

The pivotal regulatory role of non-coding RNAs (ncRNAs) in every biological process is well established and consolidated, and so it is for the process of cardiac fibrosis. In the last years, many ncRNAs, mainly microRNAs (miRNAs), long non-coding RNAs (lncRNAs) and more recently also circular RNAs (circRNAs), have been discovered as fine tuners of cardiac fibrosis, paving the way to their therapeutic targeting. Indeed RNA-targeting therapeutics have a high potential for the treatment of several diseases.

LncRNAs contribute to cardiac diseases, including fibrosis. One of the first lncRNAs reported to be dysregulated during chronic cardiac remodelling is the Maternally Expressed Gene 3 (Meg3), which was found specifically expressed in CFs and downregulated during late cardiac remodelling in mouse hearts after TAC [131]. Meg3 was known to act as a transcriptional regulator of gene expression, recruiting and guiding chromatin remodelling complexes to target sites [132, 133]. In 2017, it was identified as a regulator of the expression of the fibrosis-related gene matrix metalloproteinase-2 (MMP2) in CFs through the recruitment of p53, this latter induced by TGF-β signalling; moreover, Meg3 silencing *in vivo *after TAC decreased myocardial fibrosis and improved diastolic function, thus revealing a new potential target for the prevention of pathological remodelling in heart diseases [131].

In 2019 Hao *et al*. [134] identified the lncRNA AK137033, named Safe (Sfrp2 antisense as fibrosis enhancer), specifically enriched in the nuclei of CFs. Its expression resulted up-regulated upon both MI induction in mouse hearts and TGF-β treatment of CFs *in vitro*. Safe silencing in CFs also prevented cell proliferation, myofibroblast differentiation, and ECM proteins secretion. In addition, *in vivo* knockdown of this lncRNA in a mouse model of MI reduced the infarcted area and cardiac fibrosis, leading to the improvement of cardiac function. Safe was found to exert its pro-fibrotic effect mainly via inducing the expression of its neighbouring protein-coding gene secreted freezled related protein 2 (Sfrp2), which was proven to synergically act with Safe and the RNA-binding protein HuR in the induction of fibrosis-related genes in CFs [134].

The Nuclear Enriched Abundant Transcript 1 (NEAT1) is a lncRNA involved in cancer and some fibrosis-related diseases. Its expression was found elevated in patients with heart failure. Neat1 acts as a pro-fibrotic lncRNA, by inducing Smad7 promoter methylation through recruitment of enhancer of zeste homologue 2 (EZH2). Silencing of NEAT11 significantly alleviated the progression of cardiac fibrosis and dysfunction in a mouse model of heart failure, while overexpression of Neat1 had the opposite effect. This suggests that NEAT11 could be a good candidate for targeted antisense therapy [135].

The scaffold attachment factor B interacting lncRNA (SAIL) was shown to be reduced in cardiac fibrotic tissue and activated CFs. *In vitro* overexpression of SAIL decreased proliferation and collagen production of CFs, and could alleviate cardiac fibrosis in a mouse model of cardiac fibrosis due to MI. SAIL blocks the access of the scaffold attachment factor B (SAFB) to RNA pol II and reduces the transcription of fibrosis-related genes. The human conserved fragment of SAIL (hSAIL) could also suppress the proliferation and collagen production of human CFs [136].

The lncRNA taurine upregulation gene 1 (*TUG1*) was found to be overexpressed after MI, and this correlated with reduced levels of miiR-133b in rat models of myocardial fibrosis. Also, angiotensin-treated cardiac myofibroblasts revealed increased expression of TUG1. In addition, TUG1 knockdown prevented myofibroblast activation, while its overexpression increased cell proliferation and collagen production. Authors proved TUG1 could act as a sponge against miR133b in CFs, thus promoting the expression of CTGF, a target of miR133b. On the basis of this evidence, the axis TUG1/miR133b/CTGF could be a new potential target for the treatment of cardiac fibrosis [137].

### 5.3 Micro-RNAs

Several miRNAs play a central role in the regulation of cardiac fibrosis. MiR-21 is highly expressed in cardiac cells, preferentially in non-myocytes, and it is known to be up-regulated and involved in the development of cardiac fibrosis, mainly promoting the activation of the ERK-MAPK pathway in CFs [138]. MiR-21 inhibition has been already proven effective for the prevention of cardiac fibrosis and dysfunction in small animal models of heart injury [139, 140]. Recently, Hinkel *et al*. [141] have tested the feasibility and anti-fibrotic efficacy of miR-21 silencing also in large animal models of heart failure, laying the foundation for potential applications in humans. In detail, the local, intracoronary delivery of locked nucleic acid (LNA)-antimiR-21 in pig hearts after I/R injury led to a reduction of adverse myocardial remodelling, associated with decreased fibroblast proliferation and macrophage infiltration, as well as improvement of cardiac function [141].

A critical miRNA in cardiac fibrosis is miR-125b, which has been proven to be necessary and sufficient for fibroblast-to-myofibroblast transition. MiR-125b expression was found to be enhanced in failing human hearts and in various animal models of cardiac fibrosis, as well as in CFs stimulated with TGF-β. Mechanistically, miR-125b targets the 3′-UTR of Apelin, a repressor of the fibrogenic pathway acting on the angiotensin II/TGF-β axis, thus promoting myofibroblast differentiation and activation. Indeed, *in vivo* miR-125b silencing through LNA delivery caused the attenuation of angiotensin-induced interstitial fibrosis in murine hearts [142]. In addition, also p53 was found to be a target of miR-125b, which induced in this way fibroblast proliferation. An in-depth analysis also revealed that miR-125b was at the centre stage of the fibrosis signalling, regulating many other related pathways, therefore representing a valid new potential target for anti-fibrotic therapies.

Another regulator of fibroblast-to-myofibroblast transition was identified in miR-146b-5p, which is overexpressed in the blood of patients with myocardial ischemia, and in the heart of mice with MI. Interestingly, miR-146b-5p is up-regulated in CFs, endothelial cells, and macrophages by hypoxia-induced NF-κB, independently from the classical TGF-β pathway, thus driving the switch toward a myofibroblast phenotype. *In vivo* treatment with miR-146b-5p antagomir markedly reduced cardiac fibrosis and improved myocardial function in both murine and porcine models of MI [143].

In contrast, a miRNA with anti-fibrotic properties with a potential therapeutic application is miR-101. This miRNA was found to be down-regulated in the peri-infarct area of rats undergoing coronary artery ligation, as well as in CFs stimulated with angiotensin II. On the contrary, *in vitro* overexpression of miR-101 suppressed fibroblast proliferation and ECM remodelling protein production, mainly targeting c-Fos, a downstream factor of the TGF-β signalling. The induction of miR-101 overexpression *in vivo* in post-infarct rat hearts remarkably alleviated interstitial fibrosis and improved cardiac performance [144].

### 5.4 Circular RNAs

Circular RNAs have been involved in the progression of both cancer and cardiovascular diseases. Given their peculiar secondary structure, with a covalent link bridging each end of the linear RNA which is converted into its circular form, circular RNAs are the most stable RNAs. This feature makes them optimal targets in RNA therapeutics.

In a rat model of post-ischemic myocardial fibrosis, circHNRNPH1 expression was found increased specifically in CFs. The expression of circHNRNPH1 could restrict the differentiation of CFs into myofibroblasts by de-repressing TGF-β signalling. The anti-fibrotic action of circHNRNPH1 is exerted through sponging miR-216-5p. This allows SMAD7 de-repression and the degradation of TGF-β receptor 1. Given the role of circHNRNPH1 in the TGF-β-mediated CF activation, this circRNA could be used to design new therapies for cardiac fibrosis linked to all cardiovascular diseases [145].

Similarly, circNFIB (which is conserved un humans) was found to be decreased in mouse cardiac samples post-MI, and its overexpression could attenuate CF proliferation [146]. Its action is dependent on TGF-β stimulation, and is mediated by the ability to sponge miR-433. circNFIB showed an anti-fibrotic effect both *in vivo* and *in vitro* on CFs, thus making it another good candidate for RNA-based anti-fibrotic therapies.

The circHIPK3 expression markedly increased in CFs and heart tissues in a mechanism mediated by TGF-β, and initiated by treatment with angiotensin II. Homeodomain-Interacting Protein Kinase 3 (CircHIPK3) was shown to promote CF proliferation, migration, and subsequent tissue fibrosis by sponging miR-29b-3p. This determined upregulating of its target genes actin alpha 2 (*ACTA2*), *COL1A1* and *COL3A1*. The silencing of circHIPK3 attenuates CFs activation by inhibiting proliferation, migration, and α-SMA expression. Interestingly, a synergistic action of miR-29 overexpression and circHIPK3 silencing showed anti-fibrotic effects *in vivo * [147].

A recent study on patients with cardiac hypertrophy showed a significant decrease in yet another circRNA, that is the circYap isoform [148]. In a mouse model of hypertrophy, the expression of circYap was negatively correlated with the expression of fibrosis genes. The restoration of circYap via ectopic expression improved heart function and cardiac fibrosis in a pressure-overload mouse model of hypertrophy. CircYap expression could directly affect both survival and cell morphology of fibroblasts *in vitro * [148].

Finally, the circ_LAS1L was found to be down-regulated in acute MI patients and CFs in another clinical study. The circ_LAS1L functional role is to sponge miR-125b, thereby de-repressing this miRNA target genes involved in activation, proliferation, and migration of CFs [149].

## 6. Biotechnological Strategies

The continuous advancements in medical and biotechnological research have led to the development of innovative approaches based on biological and biotechnological therapies designed to counteract cardiac fibrosis. They include the use of biological non-cellular products, in situ reprogramming strategies, and immunotherapy approaches.

### 6.1 Exosomes

Several adult cell populations have been studied as therapeutic and/or regenerative products for the injured heart, including selected resident cardiac mesenchymal populations. Cardiosphere-Derived Cells (CDCs) have been shown to have beneficial effects, mainly through a protective paracrine action [150, 151], mediated in part by the release of extracellular membrane vesicles (EMVs), such as exosomes [152]. Human and rat dermal fibroblasts were treated with cardiosphere-derived EMVs (CSp-EMV) and then injected into rat hearts subjected to left anterior descending artery ligation [153]. *In vitro* treated fibroblasts displayed a less fibrotic phenotype, with significantly reduced levels of fibroblast specific protein 1 (FSP1) and discoidin domain receptor 2 (DDR2), and showed increased secretion of pro-angiogenic factors, such as stromal-cell derived factor 1 (SDF-1) and vascular endothelial growth factor (VEGF). Moreover, they exerted pro-angiogenic and anti-apoptotic effects *in vitro*. The *in vivo* administration of exosome-primed fibroblasts attenuated adverse remodelling, reduced the infarct area, and enhanced cardiac function. Authors speculated this therapeutic activity could be due to the specific profile of miRNAs carried by the EMV, since a similar effect on cardiomyocytes was previously proven for exosomes from CDCs [152]. This is an interesting example of a biological product directly influencing the functional phenotype of fibroblasts, with a relevant therapeutic outcome.

Recently, also the curative effect of exosomes derived from cortical bone stem cells (CBSCs) has been tested in murine models of I/R injury. CBSCs were already proven to exert beneficial effects, in terms of decreased scar size and improved cardiac function, when injected in mouse and pig models of MI [154]. The same effects were recapitulated in I/R mouse hearts where CBSC-derived exosomes (CBSC-dEXOs) were administered, highlighting their key role in mediating cardio-protection [155]. Mechanistically, *in vitro* treatment of CFs with CBSC-dEXOs decreased the expression of pro-fibrotic genes, such as collagen I, collagen III, and MMP2, even to a greater extent than whole CBSC-conditioned medium from which the exosomes were isolated. In addition, CBSC-dEXOs were also proven to significantly reduce CF activation induced by TGF-β. After analyzing the RNA content of CBSC-dEXOs, the authors suggested that this anti-fibrotic role could be exerted by small non-coding RNAs, mainly miRNAs: in fact, they caused the downregulation of ncRNAs implicated in ribosome stability and protein translation of factors necessary for the fibroblast-to-myofibroblast transition.

### 6.2 Direct Reprogramming

Cardiac regeneration aims at restoring functional cells that have been lost in the damaged heart. A therapeutic option to recover lost cardiomyocytes is the direct reprogramming of fibroblasts into cardiomyocytes through genetic tools or small molecules. This strategy could be also viewed as a way of simultaneously replacing potentially fibrotic cells with new parenchymal cells, thus representing also an anti-fibrotic approach. Several strategies have been proposed for the delivery of direct reprogramming factors in recent years, and we briefly summarize a selection based on artificial vectors/carriers.

Mónica P. A. Ferreira *et al*. [156], developed a functionalized spermine-modified dextran-based (AcDXSp) nanoparticle that allows the release of two molecules—CHIR99021 and SB431542—capable of inducing fibroblast reprogramming. Further functionalization of the nanoparticles with atrial natriuretic peptide (ANP) allowed tropism towards cardiac cells [157]. Specifically, AcDXSP nanoparticles were incubated with non-myocytes, and the effect of the release of the active molecules was confirmed. In fact, the administration of CHIR99021 significantly enhanced β-catenin stabilization, increasing its levels in both cytoplasm and nucleus. Instead, the release of SB431542 (a TGF-β inhibitor) prevented the intracellular translocation of SMAD3 to the nucleus, and inhibited its phosphorylation by anaplastic lymphoma kinase (ALK) [158]. This evidence thus supported the efficacy of this nanoparticle-based system for the direct reprogramming of fibroblasts into cardiomyocytes as a potential cardiac therapy [156].

Poly (lactic-co-glycolic acid) (PLGA)-polyethyleneimine (PEI) nanocarriers encapsulated with microRNAs represents a low-toxicity system to induce the direct reprogramming of cardiac human fibroblasts into cardiomyocyte-like cells. These nanoparticles were loaded with miR-1 and miR-133a, that initiate and drive muscle development and differentiation [159, 160]. Direct fibroblasts reprogramming was evaluated by the expression of markers such as cardiac troponins and α-actinin, confirming this strategy as a valid method for *in vivo* delivery and fibroblast targeting [161].

Qiaozi Wang *et al*. [162] have developed another non-viral system to reprogram CFs in situ, by coating neutrophil-mimicking membranes on mesoporous silicon nanoparticles (MSNs) with the FH peptide, and loading them with miR-1, -133, -208, and -499 (miR-Combo). Homing of the nanoparticle to the injured heart was achieved by the natural inflammation-homing capacity brought by neutrophil membranes, while the FH peptide’s high affinity to tenascin-C (TN-C), specifically expressed by CFs in the injured hearts, granted cell tropism. This study reported the intravenous injection of the nanoparticles loaded with the miR-combo in a mouse model of myocardial I/R, and evaluated the efficient in situ reprogramming of CFs into cardiomyocyte-like cells. Despite low rates of reprogramming, this strategy of delivering showed nonetheless an improvement in cardiac function, and attenuation of fibrosis *in vivo * [162].

### 6.3 CAR-T

Recently an innovative strategy based on chimeric antigen receptor T-cells (CAR-T) was proposed as an additional method to reduce cardiac fibrosis. The gene signature of CFs derived from healthy versus diseased human hearts were analysed, and fibroblast activation protein (FAP) was found as a specific marker of activated fibroblasts which is not expressed in cardiomyocytes. The study suggested that the adoptive transfer of antigen specific CD8+ T cells targeting the FAP protein can lead to partial removal of activated CFs *in vivo*, with significant reduction of cardiac fibrosis and restoration of function after injury in mice [163]. Joel G Rurik *et al*. [164] developed a therapeutic approach to generate transient CAR-T cells directly *in vivo* by delivering modified messenger RNA (mRNA) in T cell–targeted lipid nanoparticles (LNPs). Specifically, they injected CD5-targeted LNPs in a mouse model of heart failure, favouring the delivery of modified mRNA encoding for CAR in T lymphocytes, thus producing CAR-T cells *in vivo*. The transient CAR-T cells generated were specific for the FAP protein, so their action reduced the abundance of activated fibroblasts and consequently fibrosis, also restoring cardiac function after injury [164].

## 7. Conclusions

The quest for novel therapeutic strategies against cardiac fibrosis has taken multiple promising directions, that have provided encouraging preclinical data in recent years. A key obstacle in finding one good therapeutic option for treatment of myocardial fibrosis is represented by the complexity of the signalling network involved in the activation of repair mechanisms, which includes responses to surrounding stress and cell death signals, as well as the powerful crosstalk with the immune cells compartment. Therefore, any novel therapeutic approach will have to deal with, and possibly overcome, all the opposing signals in the microenvironment of the injured myocardium, making the quest quite complex. Moreover, different aetiologies and/or pathogenetic stimuli may be differentially sensitive to the same strategy, therefore future studies will have to define also the specifics of the population of potential target patients. Finally, given these many levels of complexity, future developments of therapies might work best with a synergistic approach, where multiple pathways are targeted by the same molecule or by combined treatments.
